# Aftermath Türkiye’s double earthquake: detailed analysis of fracture characteristics and acute management from a level I trauma center

**DOI:** 10.1186/s13049-024-01217-x

**Published:** 2024-05-10

**Authors:** Mehmet Yiğit Gökmen, Mesut Uluöz, Özhan Pazarcı, Osman Çiloğlu, Hasan Orkun Varmış

**Affiliations:** Department of Orthopedics and Traumatology, University of Health Sciences, Adana City Training and Research Hospital, Adana, Türkiye Turkey

**Keywords:** Earthquake, Fracture, Bone, Injuries, Surgical treatments, Disaster medicine

## Abstract

**Background:**

This research investigated surgical interventions for the treatment of extremity and pelvic fractures and aimed to provide an analysis of management challenges under crisis conditions in a Level I Trauma Center after Türkiye’s February 6, 2023, earthquakes.

**Methods:**

The study was a retrospective examination of the medical records of 243 fracture cases associated with the earthquakes. The age, gender, time of admission, types of extremity and pelvic fractures, anatomical localizations, and surgical treatment methods for fractures were recorded. The results of these parameters were evaluated in detail, together with the results of other surgical treatments performed in the hospital in the first week after the disaster, such as fasciotomy, amputation, and wound debridement.

**Results:**

Most of the 243 (119 males and 124 females) patients with extremity fractures and pelvic fractures receiving surgical treatment were adults (*n* = 182, 74.9%). The most common lower extremity fractures among all fracture cases were tibial shaft (30.8%) and femoral shaft (20.6%) fractures. A total of 33 patients had surgical procedures for the treatment of two or more significant bone fractures involving either the extremity or the pelvic ring. The analysis showed that the median age of patients who underwent surgery due to extremity and pelvic fractures was 36 years, with a range of 1 to 91 years, which was statistically increased compared to patients who received surgery for other musculoskeletal injuries such as fasciotomy, amputation and debridement (*p* < 0.001).

**Conclusion:**

Fractures were one of the most common musculoskeletal injuries in the first days after earthquakes, and the management of fractures differs significantly from soft tissue injuries and amputation surgeries as they require implants, special instruments, and imaging devices. The delivery of healthcare is often critically impaired after a severe earthquake. Shortages of consumables such as orthopedic implants, power drills, fluoroscopy equipment, and the need for additional staff should be addressed immediately after the earthquake, ideally by the end of the first day.

## Introduction

Two major earthquakes occurred on February 6, 2023, in the province of Kahramanmaraş, which is located in a seismic zone with a high risk of earthquakes in the Mediterranean area of Türkiye. The first seismic event occurred at 04:17 in the local time zone within the Pazarcık district with a magnitude of 7.8Mw. A second one struck the Elbistan area at 13:24 local time with a magnitude of 7.6Mw. The quakes impacted a total of 11 provinces and they were felt across a vast expanse of Türkiye, covering Southeastern Anatolia, Eastern Anatolia, Central Anatolia, and Mediterranean regions [1].

**Fig. 1:**
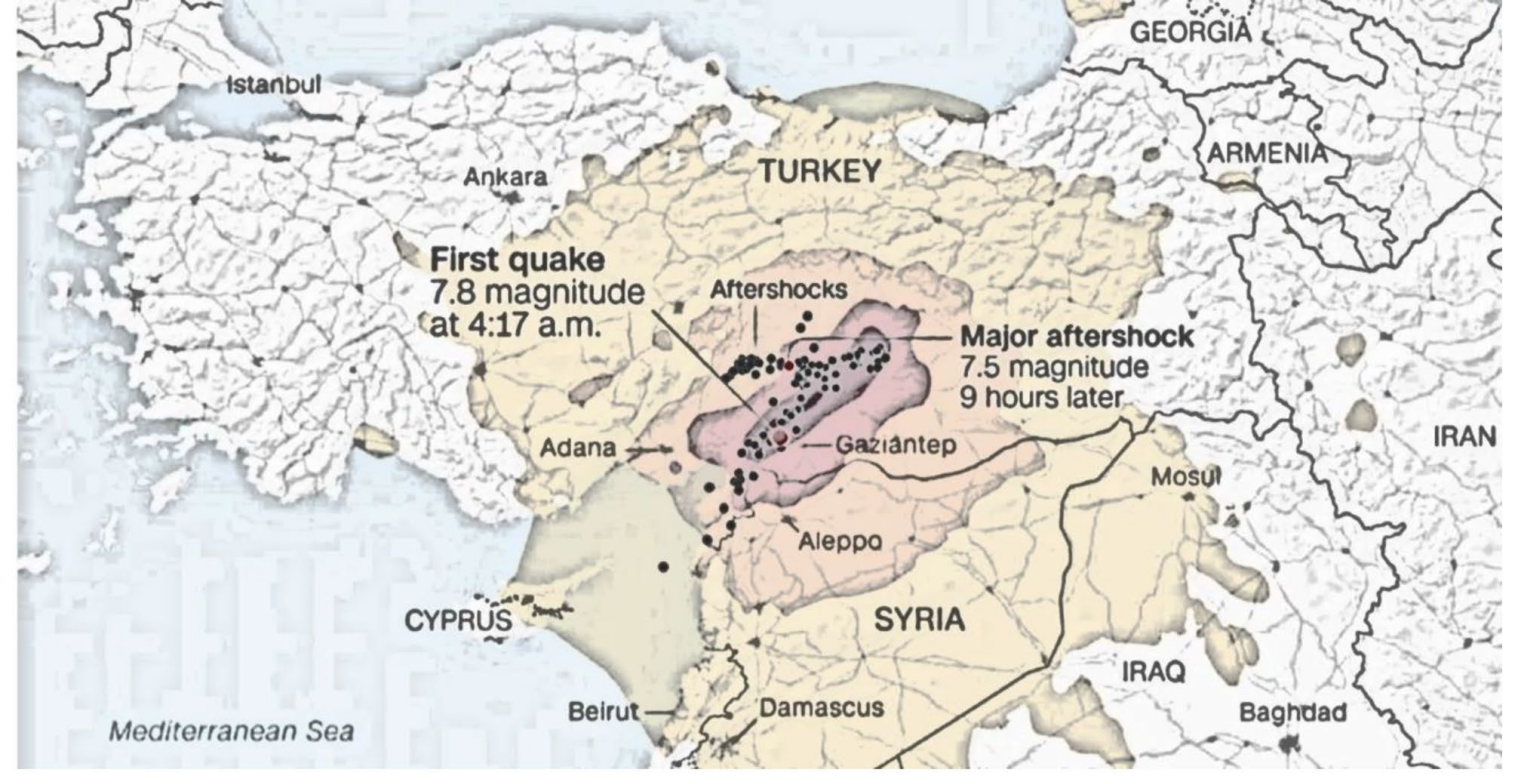
The location of Adana province on the map; the impact zone of the quakes occurred in Pazarcık (at 04:17 a.m.) and Elbistan (at 01:24 p.m.) area

The government activated the Turkish Emergency Management Plan (TEMP), issued a level four alert, and declared a state of emergency for the region with approximately 15 million people residing. Based on official statistics, the recorded number of fatalities exceeded 53,000, while the number of injured survivors was over 100,000. The area suffered significant damage to hospitals, roads, and transport infrastructure [2]. In the first three days, the majority of the injured victims were sent to healthcare facilities in nearby provinces. Adana, one of the affected provinces, suffered relatively low damage (Fig. 1).

At the time of the earthquakes, Adana was already placed on top among the 11 provinces in terms of healthcare capacity (4,345 hospital beds, 3686 physicians, and 10,082 other healthcare professionals for a 2,200,000 population). Regarding the impact of the burden of the earthquakes, Adana was relatively less affected because, in both numbers, the death toll and the number of injuries, Adana was the fifth province with 454 deaths and 7,450 injured due to the quakes [2, 3].

The Adana City Training and Research Hospital (ACH) is the region’s biggest healthcare facility, a Level I Trauma Center, and offers advanced healthcare at a tertiary level with 1550 beds. Notably, the hospital is equipped with a helipad, allowing for efficient transportation of patients in critical condition. In the aftermath of the earthquakes, ACH did not suffer any structural damage, increased its capacity, and received most of the incoming referrals.

The injury types of the victims included musculoskeletal injuries, including burns, frostbite, lacerations, traumatic limb losses, bone fractures, and crush injuries to the extremities. From an orthopedics view, among the survivors, fractures were prevalent and commonly were presented in the diaphyseal area of the femur and tibia [4].

This research investigated surgical interventions for the treatment of extremity and pelvic fractures in turbulent circumstances immediately following the earthquakes, aimed to provide an analysis of the challenges faced during the management and offer potential solutions that might be useful in overcoming the complex and challenging conditions.

## Methods

The study involved the retrospective examination of the medical records of earthquake survivors of all ages admitted to the Adana City Training and Research Hospital during the period from February 6, 2023, to February 13, 2023. In the dataset, age, gender, time of presentation, fracture types, anatomical localizations and surgical treatment methods of patients who were surgically treated for extremity and pelvic fractures were recorded. The results of these parameters were evaluated in detail together with the results of other surgical treatments performed in the hospital in the first week after the disaster, such as fasciotomy, amputation and wound debridement.

### Ethical approval

Ethical permission was obtained from the Adana City Training and Research Hospital Clinical / Human Research Ethics Committee for this study date on May 11, 2023, and decision number 2548 and Helsinki Declaration rules were followed to conduct this study.

### Statistical analysis

All data were statistically analyzed using SPSS-16 software (Statistical Package for the Social Sciences, SPSS Inc, Chicago, Il, USA). Continuous variables were shown as median (Min-Max). Mann Whitney U Test and Chi-square statistical test were used to evaluate qualitative and quantitative variables, respectively. In this study, *p* < 0.05 was set as statistically significant.

## Results

### Earthquake victim admissions in the ER

**Fig. 2:**
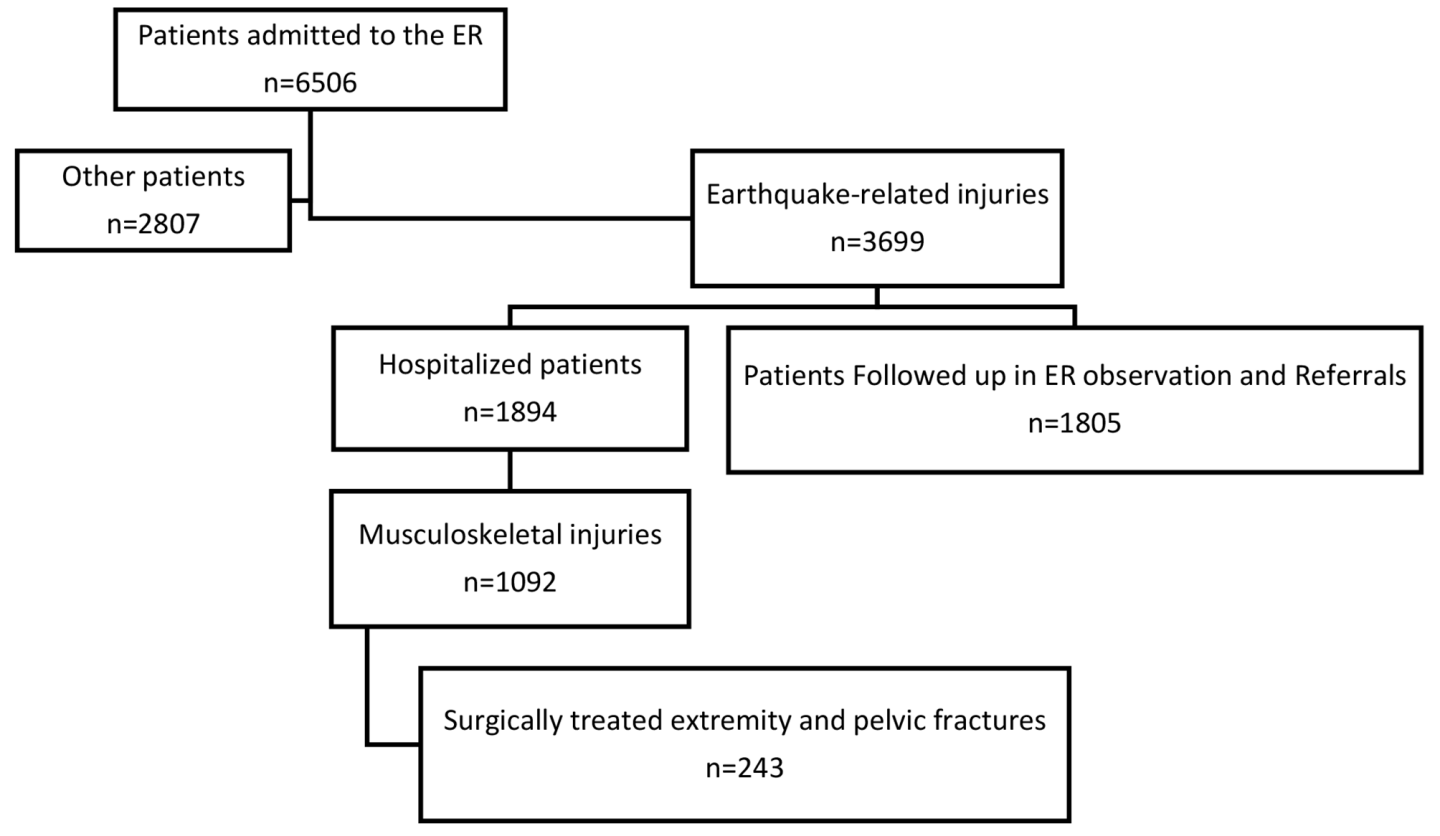
The distribution of earthquake victim admissions in the ER in the first week

During the first week following the earthquakes, 6506 victims were admitted to the Emergency Room (ER). A total of 1,092 people were admitted to the hospital due to musculoskeletal injuries associated with the earthquake. Among the cases, 827 (75.7%) patients underwent orthopedic surgery. There were 421 (50.9%) males and 406 (49.1%) females. The ages of the victims ranged from 0 to 93 years, with a median of 32 years. ER admissions of the earthquake victims during the first week are shown in Fig. 2.

### Initial orthopedic evaluation of patients with extremity and pelvic fractures

The initial evaluation and treatments for patients presenting with extremity fractures were administered in the emergency room settings. Splints and arm slings were applied to patients with closed extremity fractures who were queued for surgery, while the cases with open extremity fractures were administered tetanus vaccine or immunoglobulin, antibiotic prophylaxis, wound irrigation, sterile dressing, and splints.

**Fig. 3:**
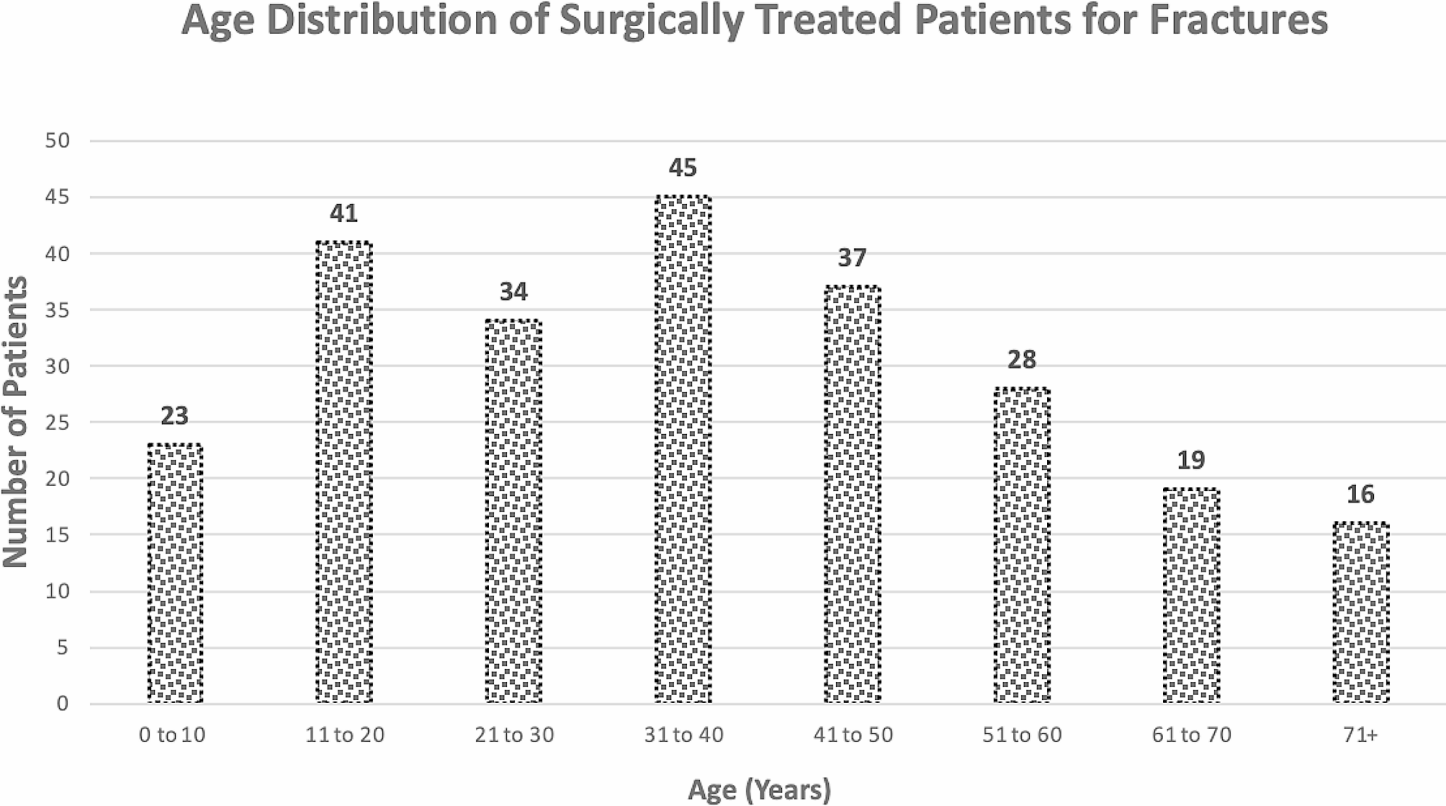
The age distribution of the surgically treated patients with extremity and pelvic fractures

The majority of 243 (119 males and 124 females) patients with extremity fractures and pelvic fractures receiving surgical treatment were adults (*n* = 182, 74.9%). The age distribution of the surgically treated patients with extremity and pelvis fractures is presented in Fig. 3.

Among all fracture cases, eight of the upper extremity fractures were open fractures, including two humerus, three forearm, and two hand fractures. Twenty-three of the lower extremity fractures were open fractures, of which five were located on the femur, 17 on the tibia, and three on the foot. There were no open fractures of the pelvic ring.

**Fig. 4:**
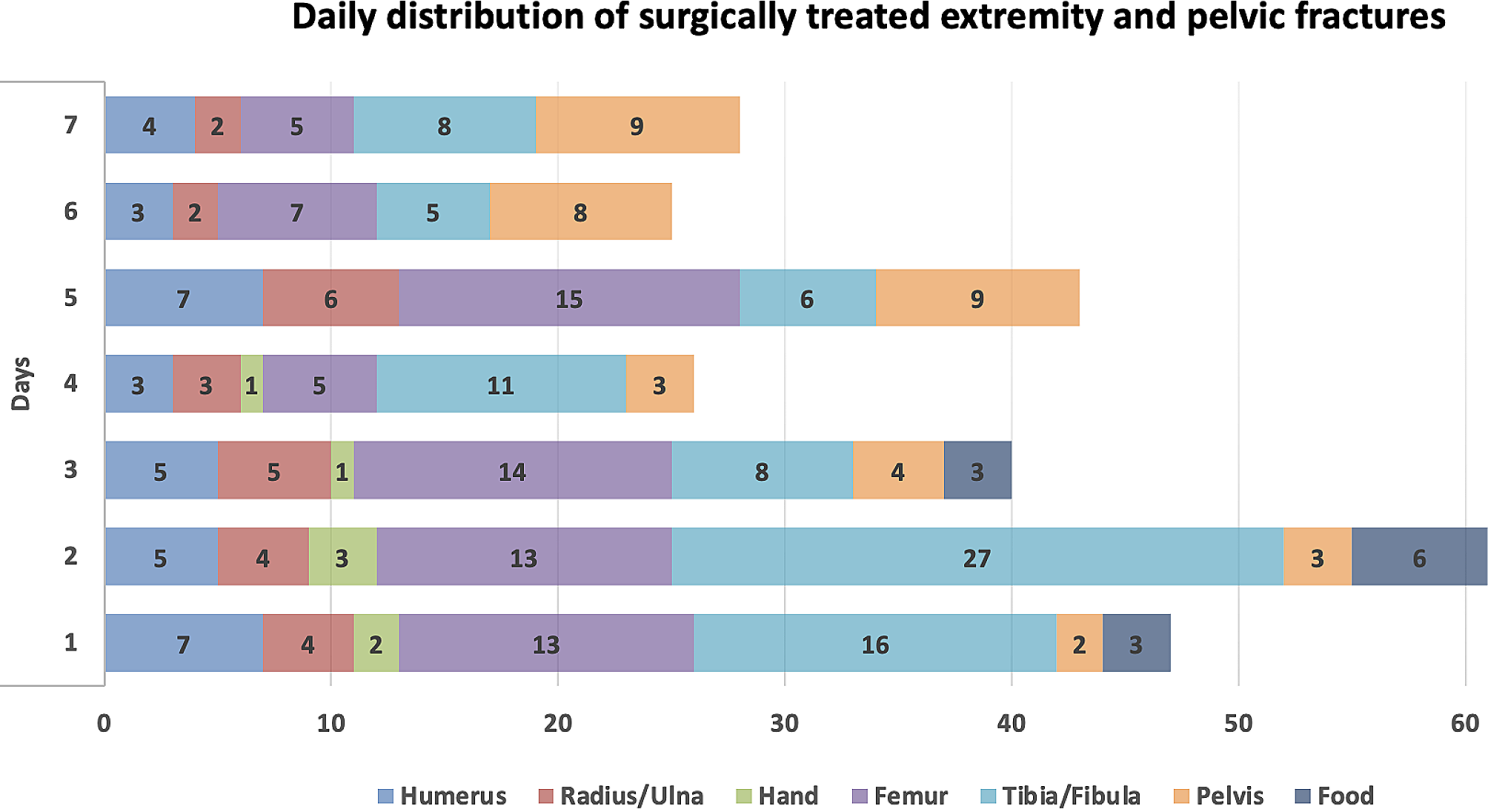
Daily distribution of the number of surgically treated extremity and pelvic fractures

The analysis showed that the median age of patients who underwent surgery due to fractures was 36 years, with a range of 1 to 91 years, which was statistically increased compared to patients who received surgery for other musculoskeletal injuries (*p* < 0.001). The daily number of fractures surgically treated for extremity and pelvic fractures for the first week is presented in Fig. 4.

### Acute management and surgical treatment of extremity and pelvic fractures

Adana City Training and Research Hospital has over 7,000 employees and 1,500 beds. There were eight lecturers, 15 orthopedic physicians, and 24 resident doctors at the time of the earthquake assigned to the Orthopedics and Traumatology Clinic of the hospital.

We used a social networking platform WhatsApp (Meta Platforms, Inc. ® ATTN/CA, USA) to create groups to track emergency patient admissions, preoperative and postoperative patients with fractures, and patients in inpatient wards, and these new technologies played a critical role in facilitating communication.

Although the social networking platforms were in use and the communication among the personnel was dramatically increased, the high volume of patients overwhelmed the clinic staff. On a regular workday, there were six operating rooms allocated for the orthopedics clinic, but suddenly became insufficient under post-earthquake circumstances, and the number of rooms was increased to 15. Also, the usual “working hours” term was off-use, and almost all staff were required to stay at the hospital and carry on the duties for the first few days.

Fortunately, five additional orthopedic surgeons from other hospitals, selected and authorized by the Ministry of Health, were assigned on the third day and joined the ACH Orthopedics and Traumatology Clinic staff for help, and the workload per surgeon decreased. A similar staff increase was observed in all levels.

During the first three days, the ratio of the extremity and pelvic ring surgery cases among all fracture surgeries was 50%. The OT Clinic staff encountered a significant accumulation of fracture surgery cases, resulting in a lack of resources in the operating theatres. Especially, the three C-arm fluoroscopy systems assigned to the clinic failed to meet the increased demand. The surgery priority was given to cases with open fractures, fractures in the lower extremities, and injuries accompanied by crush injuries with compartment syndrome. The cases with closed fractures and upper extremity fractures in the queue for surgery were temporarily fixated by splints. The arrival of three more fluoroscopy devices resulted in an increase in the number of surgical procedures performed, thereby leading to a reduction in the concentration of patients with fractures.

In the group of patients who underwent surgical intervention for fractures, 38 (17%) had pelvic ring fractures. The most common lower extremity fractures among all fracture cases were tibial shaft (30.8%) and femoral shaft (20.6%) fractures. A total of 33 patients had surgical procedures for the treatment of two or more significant bone fractures involving either the extremity or the pelvic ring. The distribution of patients who underwent surgical treatment for fractures categorized according to the anatomical location of the fracture and fixation methods is demonstrated in Table 1.

**Table 1:** Distribution of patients who underwent surgical treatment for fractures, categorized according to the anatomical location and fixation methods of the fracture

Localization	*n*	Percentage	Plate-Screw systems	Intramedullary systems	External fixators/K Wires	Arthroplasty
Upper limb	41	16.9%				
Humerus	19	7.8%	11	1	7	-
Radius/Ulna	15	6.2%	8	2	7	-
Hand	7	2.9%	2	-	4/1	-
Lower limb	164	67.5%				
Femur	70	28.8%	16	28	13	13
Tibia/Fibula	82	33.8%	14	27	41	-
Foot	12	4.9%	1	-	10/1	-
Pelvis	38	15.6%	33	-	5	-

In addition to the fracture surgeries, fasciotomies were performed on 35 fracture patients with fractures located on the tibia/fibula, femur, humerus, radius/ulna, and hand. (21, 9, 2, 2, and one, respectively). Among the patients with musculoskeletal injuries, 10.4% of patients with fasciotomy had fractures at the same time; 38.1% without fasciotomy had no fracture (*p* = 0.0001).

Furthermore, debridement surgery was conducted on 56 patients with fractures. In 14.3% of patients with musculoskeletal injuries who underwent debridement, a fracture was also present at the same time; in 38.6% of patients who did not undergo debridement, no fracture was detected (*p* = 0.0001).

In 35 of the cases, fasciotomy was also applied in addition to the debridements; the remaining received exclusively external fixators for the open fractures. Following the cleaning of the fasciotomy sites with hypochlorous acid, vacuum-assisted closure (VAC) was applied at three-day intervals during the postoperative monitoring of patients with no indications of infection who underwent surgery for fractures for open wounds and fasciotomies. Wounds of patients who underwent surgical treatment for closed fractures were dressed daily with tincture of iodine and gauze. A wound care bandage made of paraffin antiseptic gauze was used in fracture cases accompanied by deep abrasion wounds.

During the first week, 13 (5.8%) of the patients who underwent surgical treatment for their fractures underwent transhumeral, forearm, transfemoral, knee disarticulation, and transtibial amputation surgery (*n* = 1, 1, 2, 1, and 8, respectively) (*p* = 0.547).

### Early follow-up patients with fractures and postoperative rehabilitation

The large volume of patients requiring fracture surgery increased the burden of wound care. Wound care teams were formed and assigned to each hospital block to ensure uninterrupted wound care after surgery. The teams comprised of two wound care personnel, an orthopedic specialist, and a resident physician. Due to the loss of homes in the earthquake, most patients resisted being discharged. Hence, an efficient discharge procedure did not take place. Specifically, after surgery for extremity fractures, patients whose course of treatment was finished but could not be released were moved to the physical therapy and rehabilitation wing of ACH, where physiotherapists and orthopedic specialists managed post-operative wound care and orthopedic rehabilitation. In cases without accommodations or a companion, the social services department was notified at the time of discharge.

## Discussion

The Kahramanmaraş twin earthquakes catastrophe is the fifth severe earthquake of the 21st century in terms of death toll [2].

In the earthquake zone, ACH experienced a significant increase in the number of patients admitted, especially in the first days following the severe earthquake. The increase in the number of patients can be attributed to both the scale of the disaster and the significant impairment of nearby healthcare facilities [5].

The great majority of those affected by the earthquake were admitted to the emergency department at ACH and received hospitalization during the initial 72-hour period. The high incidence may be attributed to three major factors: the hospital’s close proximity to all provinces in the earthquake zone, the possibility of sophisticated medical or surgical interventions, and the quick transportation of referrals by air ambulances facilitated by the presence of a helipad.

The rise in the number of patients seeking admission to the emergency room for problems unrelated to earthquake trauma was an additional matter of concern. The exponential increase in the number of patients in the emergency room has resulted in a reduction in both the amount of time available for treatment and the physical space available for care. Similar conditions were observed following other quakes [6, 7]. Therefore, in disasters where musculoskeletal injuries are more likely to be expected, additional care teams and physical spaces should be included in the initial management design.

In a research conducted by Rigal focusing on catastrophes, it was suggested that the simultaneous transport of a substantial number of wounded victims might lead to delays in patient care, thus decreasing the overall success rate dramatically [8]. Our use of a social networking platform (WhatsApp) to create groups to track emergency patient admissions, preoperative and postoperative patients, and patients in inpatient wards was not a first and already reported in other studies, and these new technologies were shown to play a critical role in facilitating communication [9, 10].

Musculoskeletal injuries include complex conditions like fractures, crush injuries, limb loss, burns, and frostbite, which are frequently observed among a significant portion of patients [11, 12]. According to Mulvey et al., soft tissue injuries and fractures were the most frequent types of injuries that occurred in the 2005 Kashmir earthquake [13].

There are reports documenting an overall incidence of 18% of surgically treated fractures among those injured in the 2005 Barakott earthquake in Pakistan [14]. Our study showed that 6.6% of individuals affected by earthquakes (total *n* = 3699) underwent orthopedic surgical intervention for the treatment of fractures.

Our study finding showing that the most frequently surgically treated fracture location was the tibia was in parallel to the literature findings, including the studies of Özdemir et al. indicating that the ratio of lower extremity injuries was higher compared to the upper and Mackenzie et al. presenting that a significant proportion of orthopedic injuries resulting from earthquakes were characterized by fractures, with the tibia being the most affected anatomical region. In terms of orthopedic traumas, the lower extremities are the anatomical body parts most commonly affected in geophysical disasters [10, 15].

Moreover, the research conducted by Görmeli et al. and Tahmasebi et al. presented findings that support the existence of a correlation between the occurrence time of earthquakes and the frequency of the fractures [16, 17]. The researchers concluded that in cases where the earthquake took place during the afternoon or early evening, a larger proportion of the affected individuals were awake at the time, and there was a higher incidence of distal bone injuries. Accordingly, our findings show that distal fractures were not common, and instead, shaft fractures were dominant.

Severe soft tissue damage resulting from high-energy trauma, particularly caused by natural catastrophes such as earthquakes, highlights the need for external fixation surgery as a crucial approach to preserving limb integrity. Such a surgical procedure is also recommended for those who have accompanying severe trauma-related (e.g. head or thorax trauma) additional medical conditions [18, 19].

The prompt delivery of external fixation materials to local hospitals affected by earthquakes has significant importance. The utilization of external fixation is mostly favored in the surgical management of major bone fractures due to the notable incidence of open fractures and compartment syndrome. Therefore, the transfer of medical supplies should immediately be initiated following a geophysical disaster. On the other hand, although in our study, we observed that external fixators were used for the fixation of fractures in 73 (30%) earthquake victims, Bar-On et al. reported that external fixation was used in 31% of the patients, while Phalkey et al. reported this rate as less than 2% [20, 21].

Min et al. suggested that effectively managing the considerable quantity and diverse range of limb fractures in individuals affected by seismic events is a significant obstacle as they strive to adhere to the principles of safety, efficiency, and efficacy [22]. Due to its location in the earthquake zone, ACH, as a tertiary-level hospital, faced the challenge of simultaneously prioritizing patient survival and effectively managing fracture treatments and the unique demands of the circumstances.

Concurrent with the various surgical procedures conducted, the orthopaedic implants most commonly utilized were the plate-screw and intramedullary fixation systems. While there were no significant issues with the availability of orthopaedic implants and sterilization of surgical sets, the scarcity of sterile electric drills emerged as a noteworthy concern. It is also highlighted in the study conducted by Asfuroğlu et al. that similar problems are observed in the surgical treatment of orthopaedic patients in the chaotic post-disaster period [23].

The utilization of C-arm fluoroscopy is a customary procedure in the management of musculoskeletal injuries. Certain authors have documented challenges encountered during surgical procedures as a result of the limited availability of fluoroscopy [20]. The fluoroscopy machines in our operating room could not meet the demands of the patient population during the initial days, necessitating the implementation of a queuing system based on the urgency of the patient’s condition. During this period, the medical staff immobilized the injuries using splints and began the required initial therapy within the hospital wards. The resolution of the increasing queue for fluoroscopy problem was decreasingly solved upon the arrival of three more fluoroscopy devices. Using additional C-arm fluoroscopy, we used a damage control orthopaedics method for multiple-trauma patients, shown by Morelli et al. to reduce mortality and morbidity [24].

As a tertiary care facility, Level A Trauma Center, we experienced a substantial number of patients presenting with severe crush injuries and complex fractures. Notably, there were no instances of treatment delays observed for patients.

### Limitations of the study

The study has some limitations. Firstly, it is a retrospective study based on medical records. In the context of chaotic situations, it is possible that the accuracy of patient files may have been incomplete, notwithstanding efforts made to double-check all. Furthermore, the research centered on individuals who experienced significant extremity and pelvic fractures and managed to survive the earthquake, seeking medical attention at ACH. Also, it is worth noting that the duration of the follow-up period, which involved only the first week, may not be sufficient for accurately assessing potential complications such as infection, non-union disorders, and implant failure arising from fracture treatments. Finally, the study did not provide specific information on significant organ damage and accompanying extra disorders.

## Conclusion

Fractures were one of the most common musculoskeletal injuries in the first days after earthquakes, and the management of fractures differs significantly from soft tissue injuries and amputation surgeries as they require implants, special instruments, and imaging devices. The delivery of healthcare is often critically impaired after a severe earthquake. Therefore, hospitals near the epicenter should promptly complete the necessary preparations, specifically in the operating and emergency rooms, and increase the bed capacity. Shortages of consumables such as orthopedic implants, power drills, fluoroscopy equipment, and the need for additional staff should be addressed immediately after the earthquake, ideally by the end of the first day.

## Data Availability

The data supporting this study’s findings are available from the corresponding author MYG, upon request.
